# Understanding multilingualism and its implications

**DOI:** 10.3389/fpsyg.2014.01464

**Published:** 2014-12-23

**Authors:** Mary G. O'Brien, Suzanne Curtin, Rahat Naqvi

**Affiliations:** ^1^Linguistics, Languages and Cultures, University of CalgaryCalgary, AB, Canada; ^2^Language Research Centre, University of CalgaryCalgary, AB, Canada; ^3^Psychology, University of CalgaryCalgary, AB, Canada; ^4^Werklund School of Education, University of CalgaryCalgary, AB, Canada

**Keywords:** bilingualism, language acquisition, psycholinguistic methods, language pedagogy, second langauge literacy development

The world's demographics are in a state of flux. Approximately half of the world's population is bilingual (Grosjean, 2010). Just over half of all Europeans speak a language other than the official language in a given country, and 25% of them report that they are able to hold a conversation in at least two additional languages (European Commission, 2012, p. 18). Bi- and multilingualism are also the reality in North America. Grosjean (2012) estimates that 20% of Americans are bilingual. In 2011, over 20% of Canadians reported speaking a mother tongue other than English or French, and the number of Canadians who report being bilingual is rising rapidly (Statistics Canada, 2012). While the causes of increased bi- and multilingualism vary, the repercussions of this demographic shift are wide reaching.

In August 2013 the Language Research Centre at the University of Calgary brought together a range of experts working on issues related to the acquisition of multiple languages to consider the implications of multilingualism in our society. Discussions at the conference entitled “Interdisciplinary Approaches to Multilingualism” were focused around three key areas: language acquisition, psycholinguistic research methods, and second language pedagogy and literacy development. These broad fields are represented in this issue of *Frontiers*.

## Acquisition

Barlow's contribution investigates the role of age effects in the production of English and Spanish /l/ by early and late Spanish-English bilinguals. The results, which indicate that the sound systems of both early and late bilinguals interact, add to our understanding of the complexities of acquiring multiple languages across the lifespan. Shea's (2014) response to Barlow (2014) focuses on the complexity of understanding cross-linguistic allophonic variation and on the importance of exposing learners to conditioned variability.

The research by Bak et al. (2014) is an investigation of the so-called “bilingual advantage” on attention tests. Like Barlow, the authors wish to determine the extent to which early and late bilinguals differ. The results indicate that bilinguals—regardless of age of acquisition—show certain advantages on the Test of Everyday Attention. In her response to Bak et al. (2014), Macleod (2014) focuses on the advantages and disadvantages of making use of experimental results for clinical work with bilinguals. She points out that we must determine whether experimentally significant results truly matter in clinical settings. She extends the discussion to studies of vocabulary acquisition and concludes that rigorous testing of tools and a clear understanding of the backgrounds of bilinguals are essential in order to avoid potential misdiagnoses.

Lechner and Siemund's (2014) contribution investigates the effects of bilingualism on participants' attainment in their third language (L3) English. The participants, all of whom grew up in Germany speaking another language as a first language (L1), were from a variety of L1 and socioeconomic backgrounds. An important finding in Lechner and Siemund's (2014) work is that those participants who performed best did so across their languages: the heritage language, German, and English. The authors, who view English literacy as a type of academic achievement, couch their findings in terms of the Threshold Hypothesis. In their response to Lechner and Siemund (2014), Rolstad and MacSwan (2014) offer facilitation theory as an alternative theoretical framework to the Threshold Hypothesis. They argue that literacy skills transfer across a bilingual's languages because literacy in one language facilitates literacy in additional languages.

## Methodology

The paper by Libben et al. (2014) presents a proposal for making use of Facial Profiles, a technique based on Chernoff faces, and high-density experiments in order to understand participants, perception, and production. Acknowledging the necessity to account for individual variability in reading, speaking, and listening ability among participants, Libben et al.'s (2014) contribution provides methodological tools for researchers to embrace the complexity inherent in studies of bilinguals, especially research into the mental lexicon. In their response to Libben et al. (2014), Perret and Kandel (2014) point to a common problem within psycholinguistic research generally: the difficulty of accounting for random errors that arise when researchers rely on small samples. They echo Libben et al.'s (2014) call to capture within- and between-participant variability in psycholinguistic studies.

## Learning and pedagogy

All of the papers that focus on classroom situations (Cummins, 2014; Manterola, 2014; Naqvi et al., 2014; Ntelioglou et al., 2014) point to the need to both value and draw on the linguistic resources of bilingual students. This is in spite of the fact that students' languages are traditionally separated in bilingual and immersion schools. Naqvi et al. (2014) describe the results of three case studies that investigate translanguaging within Spanish bilingual programs in Alberta. Naqvi et al. (2014) made use of a variety of tasks including dual language books, videos, and inquiry-based learning tasks with a range of students as a means of encouraging them to engage with the schools' two languages. In response to Naqvi et al. (2014), Manterola (2014) discusses possibilities for integrating learners' languages in Basque-Spanish and Basque-French bilingual schools in the Basque Country. Manterola (2014) cites research indicating that improvement in a minority language undergoing revitalization (Basque) may be correlated with improvement in both the L1 and the L3 (English).

The paper by Ntelioglou et al. (2014) focuses on the effectiveness of instructional tasks for improving the literacy skills of culturally and linguistically diverse grade three English Language Learners (ELLs) in a large Canadian city. The authors report on the benefits of making use of students' home languages in the completion of a writing project.

Cummins's contribution focuses on the implications of policies that deny bilingual students access to their store of languages in a variety of school settings including French immersion education in Canada, mainstream English and French education, heritage language education, and the education of Deaf and hard-of hearing students with cochlear implants.

In spite of differing foci, Manterola (2014), Naqvi et al. (2014), Ntelioglou et al. (2014) and Cummins (2014) propose the implementation of policies, programs, and practices that enable students to build connections across languages.

### Conflict of interest statement

The authors declare that the research was conducted in the absence of any commercial or financial relationships that could be construed as a potential conflict of interest.
